# Half of Atlantic reef-building corals at elevated risk of extinction due to climate change and other threats

**DOI:** 10.1371/journal.pone.0309354

**Published:** 2024-11-15

**Authors:** Luis Gutierrez, Beth Polidoro, David Obura, Francoise Cabada-Blanco, Christi Linardich, Emma Pettersson, Paul Pearce-Kelly, Krista Kemppinen, Juan Jose Alvarado, Lorenzo Alvarez-Filip, Anastazia Banaszak, Pilar Casado de Amezua, James Crabbe, Aldo Croquer, Joshua Feingold, Elizabeth Goergen, Stefano Goffredo, Bert Hoeksema, Danwei Huang, Emma Kennedy, Diego Kersting, Marcelo Kitahara, Petar Kružić, Margaret Miller, Flavia Nunes, Juan Pablo Quimbayo, Andrea Rivera-Sosa, Rosa Rodríguez-Martínez, Nadia Santodomingo, Michael Sweet, Mark Vermeij, Estrella Villamizar, Greta Aeby, Khatija Alliji, Daniel Bayley, Elena Couce, Benjamin Cowburn, C. Isabel Nuñez Lendo, Sean Porter, Kaveh Samimi-Namin, Tom Shlesinger, Bryan Wilson

**Affiliations:** 1 Arizona State University, Tempe, Arizona, United States of America; 2 Species Survival Commission, Coral Specialist Group, International Union for the Conservation of Nature, Gland, Switzerland; 3 Coastal Oceans Research and Development in the Indian Ocean East Africa, Mombasa, Kenya; 4 Institute of Marine Sciences, School of the Environment and Life Sciences, University of Portsmouth, Portsmouth, United Kingdom; 5 Old Dominion University, Norfolk, Virginia, United States of America; 6 Zoological Society of London, London, United Kingdom; 7 University of Costa Rica, San José, Costa Rica; 8 Unidad Academia de Sistemas Arrecificales, Universidad Nacional Autónoma de México, México, Mexico; 9 MedCoral Program, HyT Association; 10 University of Bedfordshire, Wolfson College, Oxford, United Kingdom; 11 The Nature Conservancy, Dominican Republic; 12 Nova Southeastern University, Fort Lauderdale, Florida, United States of America; 13 Department of Biology and Environmental Science, Qatar University, Doha, Qatar; 14 University of Bologna, Bologna, Italy; 15 Naturalis Biodiversity Center, Leiden, The Netherlands; 16 National University of Singapore, Singapore, Singapore; 17 University of Queensland, Queensland, Australia; 18 Spanish National Research Council, Instituto de Acuicultura de Torre de la Sal, Castellón de la Plana, Spain; 19 Departamento de Ciências do Mar, Universidade Federal de São Paulo, São Paulo, Brazil; 20 University of Zagreb, Zagreb, Croatia; 21 SECORE International, Hilliard, Ohio, United States of America; 22 Institut Français pour la Recherche et Exploitation de la Mer, Plouzané, France; 23 Center for Marine Biology, University of São Paulo, São Paulo, Brazil; 24 Coral Reef Alliance, San Francisco, California, United States of America; 25 National History Museum of London, London, United Kingdom; 26 University of Derby, Derby, United Kingdom; 27 Carmabi Foundation, Willemstad, Curaçao; 28 Ecología en la Facultad de Ciencias, Universidad Central de Venezuela, Caracas, venezuela; 29 The Hawaiʻi Institute of Marine Biology, Kaneohe, Hawaiʻi, United States of America; 30 Centre for Environmental, Fisheries and Aquaculture Science, Weymouth, United Kingdom; 31 Fauna & Flora International, Cambridge, United Kingdom; 32 Climate Change Cluster, University of Technology Sydney, Ultimo, NSW, Australia; 33 Oceanographic Research Institute, Durban, KwaZulu-Natal, South Africa; 34 Tel Aviv University, Tel Aviv, Israel; 35 University of Oxford, Oxford, United Kingdom; MARE – Marine and Environmental Sciences Centre, PORTUGAL

## Abstract

Atlantic reef-building corals and coral reefs continue to experience extensive decline due to increased stressors related to climate change, disease, pollution, and numerous anthropogenic threats. To understand the impact of ocean warming and reef loss on the estimated extinction risk of shallow water Atlantic reef-building scleractinians and milleporids, all 85 valid species were reassessed under the IUCN Red List Categories and Criteria, updating the previous Red List assessment of Atlantic corals published in 2008. For the present assessment, individual species declines were estimated based on the modeled coral cover loss (1989–2019) and projected onset of annual severe bleaching events (2020–2050) across the Atlantic. Species traits were used to scale species’ relative vulnerability to the modeled cover declines and forecasted bleaching events. The updated assessments place 45.88%–54.12% of Atlantic shallow water corals at an elevated extinction risk compared to the previous assessments conducted in 2008 (15.19%–40.51%). However, coral cover loss estimates indicate an improvement in reef coverage compared to the historic time-series used for the 2008 assessments. Based on this, we infer that, although remaining dangerously high, the rate of Atlantic reef coral cover decline has surprisingly slowed in recent decades. However, based on modeled projections of sea-surface temperature that predict the onset of annual severe bleaching events within the next 30 years, we listed 26 (out of 85) species as Critically Endangered in the IUCN Red List. Each of these species had previously been listed under a lower threatened category and this result alone highlights the severe threat future bleaching events pose to coral survival and the reef ecosystems they support.

## Introduction

The formation of the Panamanian Isthmus during the late Pliocene (~3 mya) isolated the Atlantic from the eastern Pacific [1,2]. This has resulted in there being only 83 zooxanthellate and 2 azooxanthellate shallow water corals described in this region [3,4]. All are endemic to the Atlantic Ocean basin and absent from both the Indian and Pacific oceans [4,5]. Comparing the diversity of the Atlantic to the Indo-Pacific (where there are many hundreds of species and subsequent higher functional redundancy), the loss of each species in the region will have a more direct and significant consequence for the state and functioning of the reef ecosystem [5–8]. Regional losses in coral diversity has resulted in reductions in structural complexity, carbonate budgets, and heterogenous community structures [9,10].

The Wider Caribbean (WC; Caribbean Sea, Gulf of Mexico, Florida, Bahamas, and Bermuda) has the largest Atlantic reef system, accounting for 10% of global reef coverage, and has likely operated as a source for the dispersal of species across the whole of the Atlantic, with both physical and biological barriers to connectivity playing an important role in determining current distribution ranges [2,11–14]. In contrast, much lower levels of species diversity are present in the Southwestern Atlantic off Brazil. Here corals account for as little as 0.47% of the global coral reef coverage [11,15,16]. That said, the majority of coral species which make up reefs in this region have massive growth forms and are endemic, with the Amazon and Orinoco rivers acting as a biogeographic barrier to the WC coral fauna [15,17]. The low levels of genetic and structural diversity are likely the consequence of historic changes in regional sea level during the late Holocene and the presence of a narrow continental shelf off Brazil [15,16,18].

In the Eastern Atlantic, the Mid-Atlantic Barrier impedes dispersal of species across the ocean resulting in very few coral species also occurring along the West African coast and into the Mediterranean Sea [2,17]. In fact, coral reefs are not found in the Eastern Atlantic due to a variety of regional characteristics such as low temperatures, high volumes of river discharge, and high isolation from other marine provinces. The extant corals mainly compromise azooxanthellate stony corals, with notable exceptions such as *Montastraea cavernosa* [17,19].

Over the last half-century, corals across the Atlantic have experienced severe mortality events and low recovery rates [20,21]. Steep declines in the Caribbean in particular began in the 1970s and 80s, as the western Atlantic was host to various diseases that severely impacted the structurally important Acroporid corals while also nearly driving a primary herbivore, the long spined sea urchin (*Diadema antillarum*) to functional extinction. Declines in D. antillarum, and other herbivores, have been associated with increases in algae cover with more recent populations being affected by a new epizootic event [22,23]. Many reefs remained dominated by healthy populations of the other major reef-building coral (i.e., Orbicella), yet more recent coral bleaching events caused high losses in live coral cover, especially those events that followed or occurred in tandem with diseases [24–27]. However, thermal stress and coral mortality has not been homogenous in the region with the Southern Caribbean being more severely affected by thermal events. Since 2014, the WC is being plagued by the emergence of Stony Coral Tissue Loss Disease (SCTLD). This has now been heralded as the most damaging coral epidemic in decades causing community shifts towards dominance of more weedy corals (i.e., sub-massive agaricids and *Porites astreoides*) and reductions in structural complexity and functional diversity [28–31]. Furthermore, global heating of the ocean’s surface has intensified the frequency and intensity of hurricanes in the Atlantic, effectively reducing recovery time for corals and reef calcium carbonate budgets via erosion [20,32].

The Southwestern Atlantic (Brazil), has also not escaped bleaching events (with first records dating from the early 1990s and followed by events in 1997, 2003, 2005, 2010, 2016 – 2017, and 2019) [15,33–36]. Despite the observation of bleaching, the corals in many reefs recovered quickly and therefore were reported as exhibiting a high degree of resilience to elevated temperatures [18,33]. However, there is conflicting evidence here with others showing evidence of more recent mass mortality [33]. Regardless of the thermotolerance of the corals in this region, the southwestern Atlantic has one major advantage over other areas as it is unaffected by hurricanes [15]. As for the east Atlantic, there exists little information on trends related to coral species.

These widespread threats that simultaneously operate at regional or global scales (e.g., hurricanes, bleaching and disease), can be further exacerbated by local impacts, such as coastal runoff, development, pollution, overfishing, and tourism which occur at varying degrees throughout the Atlantic [21,37,38]. The combined effects of these global and local stressors on Atlantic reef-building corals threaten the persistence of these species and the functional integrity of reef systems. However, it is well known that coral species vary in their vulnerability and resilience to such stressors yielding variation in their overall fitness, abundance, and extinction risk [39–43]. Such variability has resulted in alterations in reef community assemblages over the last several decades emphasizing the importance of regular assessments of coral populations and their subsequent conservation.

In 2008, the estimated extinction risk of the world’s known reef-building corals was published for the first time in the International Union for the Conservation of Nature (IUCN) Red List of Threatened Species. Nearly a third of all reef-building corals were threatened with extinction at the time [44], with the WC having the highest proportion (between 2 – 8%) of Critically Endangered species of all regions assessed. Here, we present and summarize the most updated extinction risk for all known 85 Atlantic reef-building corals, following a similar approach as the original 2008 assessment, but with additional consideration of the impacts of projected annual severe bleaching (ASB) events induced by rising sea surface temperatures (SST).

Estimating extinction risk plays an important role in the conservation of species globally. Assessments identify at-risk species, outlining their major threats, and as such are valuable tools in preventing continued biodiversity loss. Through reassessments, it is possible to track changes in extinction risk over time (i.e., worsening or improving conditions) that help evaluate the effectiveness of conservation interventions. For reef-building corals, continued reassessments are key in estimating the expected degree of biodiversity loss over time given the compounding threats that plague corals and coral reefs globally.

## Methods

### IUCN red list process

Reassessments for all 85 valid shallow water Atlantic reef-building scleractinian and milleporid coral species were conducted by the IUCN Species Survival Commissions’ Coral Specialist Group and more than 20 coral specialists from across the globe, all of whom contributed extensive amounts of expertise and data. This assessment evaluated all the main reef-building coral species of the North and South Atlantic Ocean (with the Mediterranean Sea), including 78 of the order Scleractinia and 7 of the hydrozoan genus *Millepora* (Order Anthoathecata). The taxonomy followed the most recent classifications from the World Register of Marine Species [3] representing 15 families and 31 genera (with two genera currently without family assignments). All species assessed are zooxanthellate (although we note, *Astrangia poculata* and *Oculina* spp. can be apozooxanthellate (either zooxanthellate or azooxanthellate). The full species list was generated in 2020 (S1 Dataset).

For each coral species, information on distribution range, population, habitat, ecology, threats, and conservation efforts were revised and updated by a group of experts and added to the species’ Red List account in the IUCN Species Information System (SIS). Following the IUCN Red List guidelines [45], species extinction risk was assessed based on the following criteria, selecting the criterion with the highest risk: Population size reduction (A), Geographic range (B), Small population size and reduction (C), Very small or restricted population (D), and Quantitative analysis (E).

**Fig 1:**
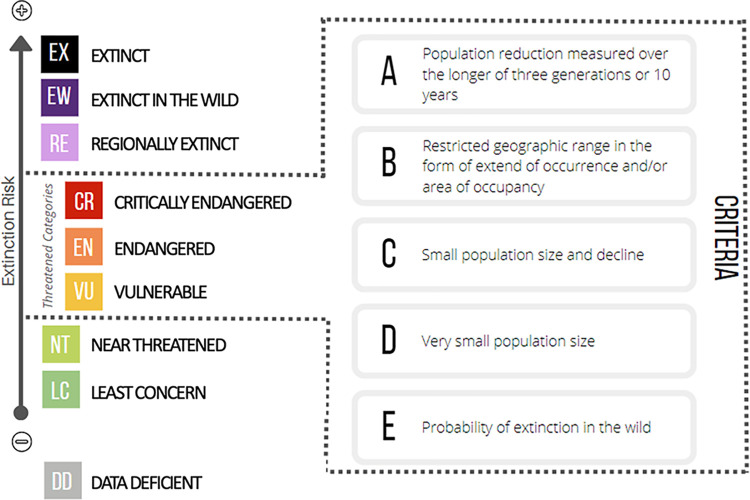
IUCN red list of threatened species categories and criteria.

The IUCN Red List of Threatened Species Criteria provides a standardized methodology for measuring relative extinction risk for a wide range of species [46]. The extinction risk of species is discretized into eight categories, ranging from Least Concern to Extinct, with an additional category of Data Deficient (DD) for cases when the information available for a species is insufficient to assess its extinction risk (Fig 1). Species assessed as either Vulnerable (VU), Endangered (EN) or Critically Endangered (CR) are considered threatened and in need of urgent conservation.

All species distributions were mapped using ArcGIS 10.7.1 and informed by known distributions in Corals of the World [4], World Register of Marine Species including World List of Scleractinia [3,47], and expert knowledge. Minimum convex polygons were created and clipped to the first 200 km off the coastline, rather than known reef habitat, to consistently map and assess corals across the entire ocean basin [48]. These included regions lacking coral reefs (e.g., Eastern Atlantic) and potential off-reef habitats. Total range size (*km*^2^) was calculated in ArcGIS as well as sub-regional range size and species richness based on Marine Ecoregions of the World [11].

Six Atlantic species not previously assessed in 2008, due to taxonomic uncertainty, have been included in this assessment update. The assessments were released on the IUCN Red List website (www.iucnredlist.org) in December 2022.

Almost all coral species were assessed under Criterion A, which measures population decline over a time period of 10 years or three generation lengths (whichever is greater). A generation length is defined as the average age of reproductive adults [45].

Criterion A is best applied to widespread species where the rate of decline exceeds the species rate of recovery [46] and can be applied to a period of three generations in the past, in the future, or a combination of past and future time frames [45]. To qualify for a threatened category, the quantitative threshold of estimated population decline begins at 30% for Vulnerable, followed by 50% for Endangered, and 80% or higher for Critically Endangered [45]. For the majority of corals, the previous assessment used an estimated average age for reproducing “adult” coral colonies of 10 years based on measures of survival and fecundity [44]. In the absence of better estimates, we retained this method, and applied a three-generation length period of 30 years.

Most coral species lack species-specific quantitative data to estimate population size and/or trends over a 30-year time frame across the majority or entirety of their global range. Where direct observational data are lacking, Criterion A allows data on habitat decline or other proxy variables that reflect ecosystem-level changes to be used as a surrogates [48]. The percentage cover of hard corals is the most widely used indicator of coral-reef ecosystem state [11] and an Essential Ocean Variable [49]. Coral cover is a direct measure of total live coral abundance and is available with global coverage updated to 2020 [11] and was used as the primary indicator for coral reef habitat decline. However, species are not evenly distributed across ecosystems and can have varying species-specific responses to ecosystem changes [50,51]. To account for this and using the same approach as the prior assessment [44], we calculated the amount of coral cover decline within each species range (over a three-generation length or 30-year period) and then adjusted this up or down based on species’ vulnerability traits, to estimate the degree of population decline for each coral species with newer surrogate datasets and updated coral species vulnerability traits.

To estimate population decline over three generation lengths in the past (Criterion A2), a surrogate dataset was used, based on the percentage of modeled coral cover loss over the past 30 years within the species range [11]. Declines projected into the future (Criterion A3) utilized the estimated average year for the onset of annual severe bleaching in the future across species’ ranges [52]. These datasets were used for all species except 11, for which sufficient species-specific data on population trends was available to assess under Criterion A (*Acropora cervicornis*, *Acropora palmata*, *Astrangia poculata*, *Balanophyllia europaea*, *Cladocora caespitosa*, *Dendrogyra cylindrus*, *Mussismilia harttii*, *Oculina arbuscula*, *Oculina patagonica*, *Orbicella annularis*, and *Orbicella faveolata*).

Three species (*Mussismilia hispida*, *Scolymia wellsii*, and *Porites colonensis*) were assessed under Criterion B due to their highly restricted range. Under Criterion B, a species is considered threatened if its global distribution range is small and there is evidence of fragmentation and/or a continued decline in habitat, population, or range size [45]. Specifically, a species must have an extent of occurrence (EOO) of less than 20,000 km^2^ or an area of occupancy (AOO) of less than 2,000 km^2^ as defined by the IUCN Red List guidelines v15 [48].

Seven other species (*Millepora laboreli*, *Oculina robusta*, *Oculina tenella*, *Oculina valenciennesi*, *Porites gabonensis*, *Schizoculina africana*, and *Schizoculina fissipara*), whose distribution ranges are poorly known, largely due to taxonomic uncertainty, were categorized as Data Deficient.

### Species vulnerability traits

For each of the 85 species, data for ten traits were compiled (Table 1) developed from the list of traits used to inform the previous Red List assessment of corals [44] and expert opinion. The traits were used as indicators of species-specific vulnerability or resiliency to overall threats. Each trait was classified into three levels of relative vulnerability: highly susceptible, moderately susceptible, and more resistant (scored as 3, 2, and 1 respectively). Overall vulnerability of a species was based on the higher between the average or mode of all ten trait scores. Species which scored high across the majority of the traits were assumed to be more vulnerable to anthropogenic threats or other stressors. Moderate susceptibility represents the uncertainty of species vulnerability given the lack of trait data and is therefore treated as the middle value between species known to be highly vulnerable or more resistant to threats. Therefore, species themselves were classified as highly susceptible (rank 3), moderately susceptible (rank 2), and more resistant (rank 1).

**Table 1:** Trait-based indicators of species relative vulnerability and resilience.

	1: More Resistant	2: Moderately Susceptible	3: Highly Susceptible
**Total Depth Range**	> 20 m	Unknown	≤ 20 m
**Dominant Depth Range**	> 10 m	Unknown	≤ 10 m
**Generalized Global Abundance**	Common	Uncommon & unknown	Rare
**Restricted or Highly Fragmented Range**	No	Unknown	Yes
**Found Off - Reef**	Yes	Unknown	No
**Disease Vulnerability**	Moderately or highly resistant	Unknown	Moderately or highly susceptible
**SCTLD**^**a**^ **Vulnerability**	Low vulnerability	Unknown/ Presumed vulnerable	High & intermediate vulnerability
**Bleaching Vulnerability**	Moderately or highly resistant	Unknown	Moderately or highly susceptible
**Recovery**	Recovers quickly	Unknown	Does not recover quickly
**Ocean Acidification Vulnerability**	Pocilloporidae	Unknown	Acroporidae, Poritidae, Merulinidae, Dendrophylliidae, Agariciidae, Astrocoeniidae and Rhizangiidae

Species traits used to rank corals on their relative vulnerability and resilience to population decline. These traits informed population reduction as modeled by the Global Coral Reef Monitoring Network coral cover data and UNEP climate projections.

^a^SCTLD – Stony Coral Tissue Loss Disease.

These vulnerability traits were collated from previous coral extinction risk assessments [44], and other assessments that related species vulnerability to depth or depth range [42,53], disease or bleaching susceptibility [43,54,55], and recovery potential [56]. Trait information and classification for each species were also informed by the expert assessor knowledge and published trait data [3,4,57,58]. Where species-specific trait data were lacking, traits were assigned a middle score of 2 (moderately susceptible) which mainly applied for traits such as bleaching, disease, and SCTLD vulnerability and recovery potential.

Species given a highly susceptible (rank 3) score were characterized by the following traits: rare abundance with shallow (≤ 10 m) and/or narrow depth intervals (≤20 m); restricted to reef habitat with a highly fragmented population, and a moderate or high susceptibility to diseases and bleaching with a low recovery rate (See Table 1 for specific traits). By contrast, more resistant species were those with traits such as: common abundance with wider depth intervals, predominantly found in waters deeper than 10 m, and that can occur in habitats other than biogenic reef. These traits also included species that were observed to be resistant to disease and bleaching, had a low susceptibility to SCTLD, and had a high recovery rate from disease and/or bleaching.

### Estimating past population trends: Coral cover loss

Estimates of coral cover loss for each species were calculated based on average coral cover changes across each species’ distribution, adjusted by species traits. For this approach, modeled changes in historic coral cover from 1978–2019, compiled by the Global Coral Reef Monitoring Network (GCRMN) [11], were used as a surrogate for estimating population decline for coral species over the past three generations (1989–2019) in eight subregions (formed from one or more ecoregion) of the Atlantic [11]. Five subregions are within the Wider Caribbean region and three are within the Brazilian region. Model outputs included the median estimate of coral cover change (50%), as well as the upper (80%) and lower (20%) uncertainty bounds or confidence intervals available for each subregion [11]. For each of these subregions (*i*), coral cover changes (*C*_*i*_) were calculated as the proportional percentage change from the starting value (*C*_0_) to the current value (*C*_1_) using the following equation:

$$C_{i}=((C_{1}-C_{0})/C_{0})\times 100$$

However, estimates of coral cover changes were not available within subregions of some coral species’ ranges, including some parts of Brazil (i.e., Amazonia, Guiana, and southeastern Brazil), the eastern Atlantic, and the Mediterranean Sea. For these subregions, coral cover loss was conservatively treated as having no change over the 30-year period and given a value of zero for all years.

Total estimates of coral cover change within each species’ range were calculated as the sum of the proportion of the species range within each subregion multiplied by the subregional coral cover percentage change over the three-generation length period (i.e., 1989–2019). Total species range (*km*^2^) was measured in ArcGIS as well as the proportion that coincides within each of the Atlantic subregions (*i*). The proportion of species’ ranges (*R*) within each subregion was multiplied by the percentage coral cover change (*C*_*i*_) for the corresponding subregion calculated from the GCRMN model and summed together for an estimation of total decline using the following equation.


$$Total\;decline=\sum \left(R_{i}\times C_{i}\right)$$

Based on species trait scores, moderately susceptible species (overall vulnerability score of 2) were assigned estimates of coral cover change from the median estimate (50%), whereas the highly susceptible (overall vulnerability score of 3) and more resistant (overall vulnerability score of 1) species were assigned coral cover changes calculated from the upper (80%) and lower (20%) bounds respectively.

### Projecting future population trends: Annual severe bleaching

For each species, future population decline was estimated based on the projected onset of annual severe bleaching (ASB) over a three-generation length time period, corresponding to 2020–2050. The onset of ASB was taken from SST projections made available by UNEP, which combined the 2019 IPCC CMIP6 global climate models and WCMC-UNEP global coral reef distribution map [52,59]. ASB describes the timeframe during which reefs are expected to experience severe bleaching events annually that result in large reductions in live coral and are not predicted to recover [60]. Heat stress for corals is quantified when sea surface temperature (SST) exceeds 1°C above the maximum monthly mean, and the indicator is cumulative during a three-month period [61,62]. ASB is expected to occur above eight Degree Heating Weeks (DHWs), which may reflect a 1°C exceedance over eight weeks, 2°C for four weeks, etc., during the 3-month window. We used the most recent global climate models, CMIP6, from which annual DHW values were calculated for 27-x-27-km grid cells that contain reef habitat (to 30 m depth) across the globe. SST projections were unavailable for the eastern Atlantic given the absence of reef habitat.

For each species, the thermal grids [52] were clipped to the species’ distribution polygon using ArcGIS with the average year of the onset of ASB estimated from the resulting grid cells. The onset of ASB was based on SST changes as projected from two CMIP6 scenarios based on Shared Socioeconomic Pathways (SSP): SSP5-8.5, potentially representing current global emissions following a precautionary approach recommended by Red List guidelines, and SSP2-4.5 representing a future reduction in emissions under current enacted climate policy [52,63,64]. Each scenario accounted for the potential adaptation of species to thermal stress. In doing so, the 1°C exceedance over the monthly maximum was increased by incremental quarter-degree Celsius intervals (e.g., between 0 – 2°C over the 1°C average monthly mean threshold) [52]. This effectively pushes back the projected onset of ASB due to adaptation potential.

The potential for adaptability to heat stress was based on species’ overall vulnerability to bleaching Table 1. Species that are estimated or known to be resistant or moderately susceptible to bleaching were inferred to have the ability to adapt to a small degree of heating. So, the estimate of ASB for these species were based on the likelihood that the species may adapt to at least 1°C above the 1°C exceedance over the monthly maximum. Species with a high susceptibility to bleaching were inferred to have a low potential for adaptation to heat stress, and therefore the 0°C quarter degree interval was applied. We assumed a higher potential for climate adaptation given higher potential for surviving heating events [65]. The coarse scale for inferred adaptability is due to the lack of a comprehensive metric to assess species-level adaptation to heat stress.

In either case, if the average year of ASB onset was projected to occur within three generation lengths (i.e., before 2050), then a projected decline of at least 80% (i.e., Critically Endangered) was assumed. In this conservative approach, a decline of at least 80% at the onset of ASB is likely an underestimation, as it has been shown corals are unlikely to survive two severe bleaching events per decade [63].

Additionally, to account for UNEP depth model limitations and potential depth refugia, if the species’ dominant depth range Table 1 extended deeper than 30 m, the proportion of the range within 0 – 30 m was multiplied by 80% to estimate the average proportional decline based on that species’ depth range. This accounts for coral species with shallow depth preferences being more frequently exposed to extreme temperatures compared to those that also occur at deeper, cooler depths [66] though there are some exceptions and corals in larger depths can have lower thermal thresholds [67,68].

### Updated method comparison

The first global extinction risk assessment of corals [44] used similar species traits but a different dataset on coral cover loss, and it did not have access to projected temperature changes. Its estimates of coral cover loss were based on expert judgement aggregated from subregions of the globe, divided into the categories “percentage of destroyed” and “destroyed plus declining” reef area [38]. It is thus not possible to directly compare the 2008 coral Red List categories with these newly updated categories. Instead, to enable an assessment of change in status over time, we used our current data and methods to back-cast coral cover changes. CMIP6 climate projections are not suitable for back-casting, as the projected DHWs are only available between 2015 – 2100, not back to 2004 – 2008, the timing of the first assessment.

The complete list of species, traits, trait scores, GCRMN declines, and back-casting estimates, average years of ASB onset at 0°C and 1°C degrees of adaptation for both the SSP5 8.5 and SSP2 4.5 scenarios, as well as the final Red List categories for all 85 Atlantic coral species are available in the Supplemental Materials.

## Results

### The extinction risk of Atlantic corals

**Fig 2:**
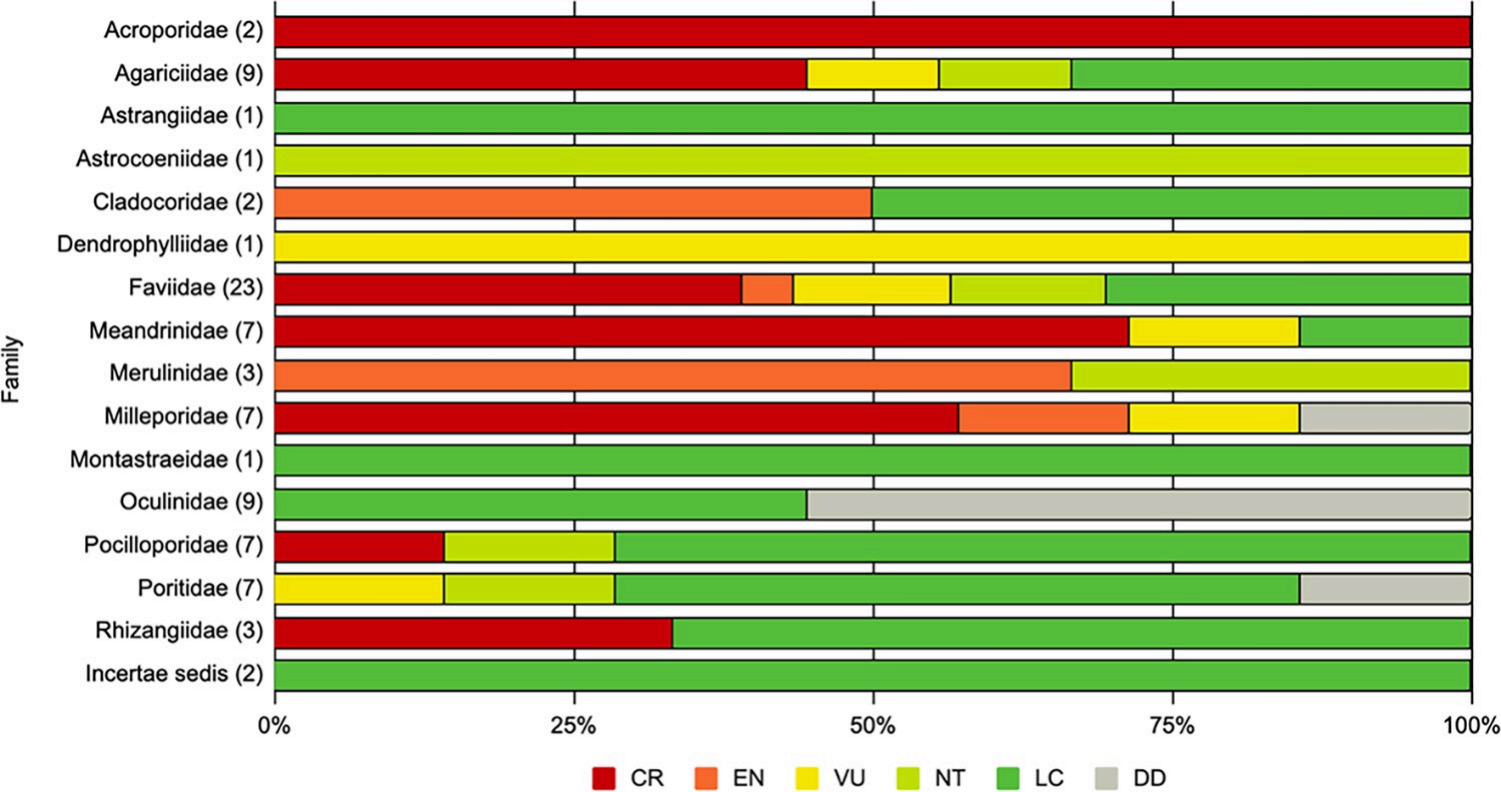
Proportion of Atlantic reef-building corals in IUCN red List categories by family. Number of species in parentheses. Red List categories are distinguished by color; Critically Endangered (CR) red, Endangered (EN) orange, Vulnerable (VU) yellow, Near Threatened (NT) green, Least Concern (LC) dark green, and Data Deficient (DD) gray.

Updated assessments for the 85 Atlantic reef-building corals for the IUCN Red List of Threatened Species were released in December 2022 on the IUCN Red List website (www.iucnredlist.org). A total of 39 species, representing 45.88% of the species assessed (*Fig 2*) are listed in a threatened category (CR, EN, or VU). The best estimate of the percentage of threatened species, accounting for Data Deficient (DD) species, is 50% (45.88% - 54.12%) of all Atlantic reef-building corals.

From the updated assessment, the coral families with the highest proportion of species threatened include; Acroporidae and Dendrophylliidae (100%), Agariciidae, Cladocoridae, Faviidae, Meandrinidae, Merulinidae, and Milleporidae (≥ 50%). There are just two species of *Acropora* in the Atlantic (*A*. *cervicornis* and *A*. *palmata*), both of which qualify for Critically Endangered (CR) status due to observed past population declines. There is also a single species of Dendrophylliidae (*Balanophyllia europaea*), endemic to the Mediterranean, which was classified as Vulnerable.

Since the 2008 assessment, six additional species have been described and are now officially recognized as distinct, increasing the number of assessed Atlantic reef-building corals from 79 to 85 species. Of the 79 species listed in 2008, 12 (15.2%) were listed in a threatened category (CR, EN, VU), two (2.5%) as Near Threatened (NT), 45 (57%) as Least Concern (LC), and 20 (25.3%) as DD [44]. When compared to the current listing, 32.9% (26 of 79 species) now have a higher extinction risk (e.g., VU to CR or LC to NT, etc.) than previously recognized. In contrast, only 3.8% (3 of 79 species) qualify for a lower extinction risk (e.g., CR to EN or VU to LC, etc.). However, it should be noted that all but one of these changes represents ‘non-genuine’ changes in listed extinction risk. A result due to the different methodologies utilized for the 2008 assessment and the current update. *Dendrogyra cylindrus* is the only species to have a ‘genuine’ change in status from VU to CR, given that both assessments have been informed by species-specific population data and not surrogate datasets. Further, most species (38% or 30 of 79 species), have maintained their previously determined extinction risk category. The extinction risk of 15 species previously categorized as DD were also assessed due to sufficient data, but five species (5.9%) of the total 85 remain DD.

### Spatial distribution of threatened corals

**Fig 3:**
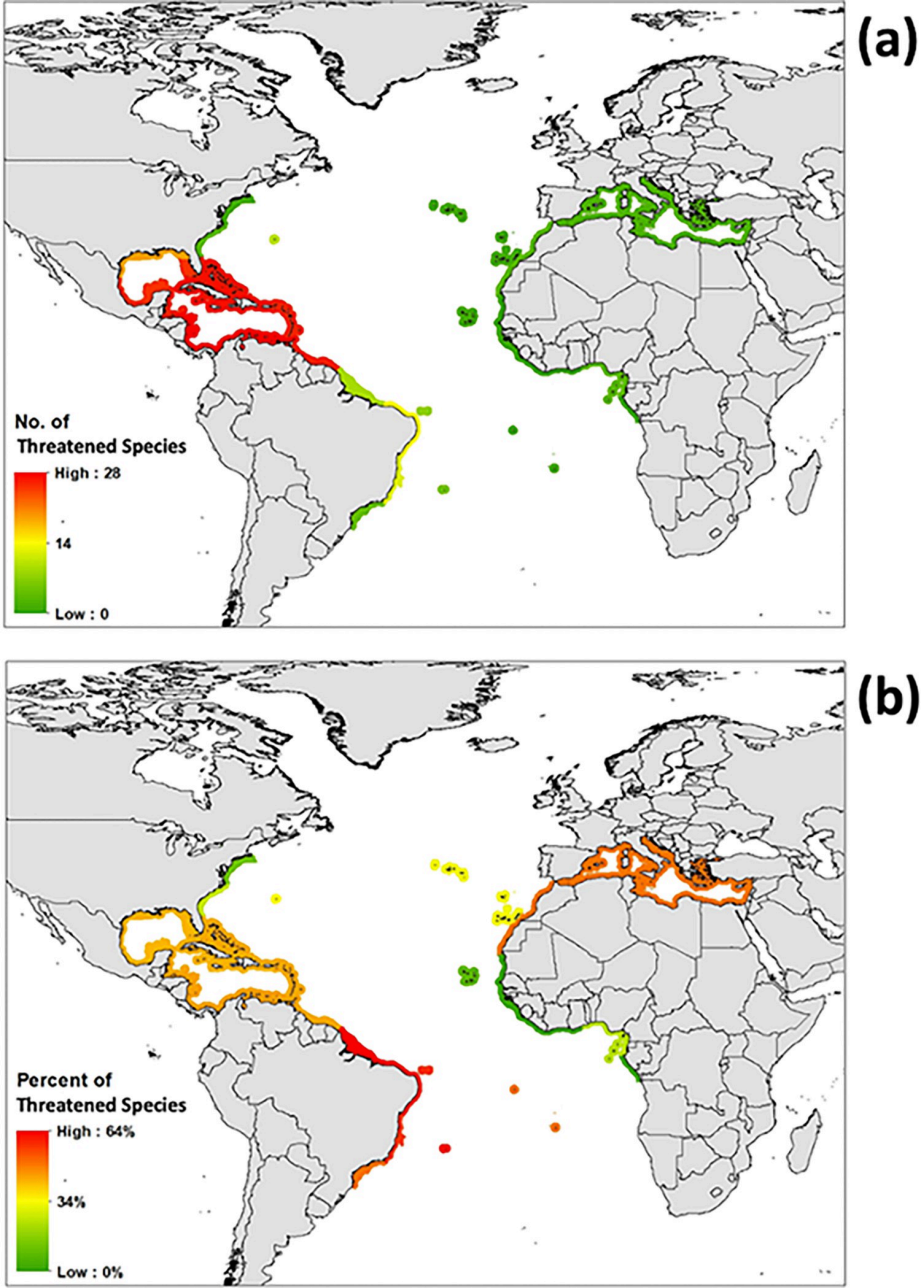
Distribution of threatened Atlantic reef-building corals by marine ecoregions. Atlantic reef-building corals as (a) the number of species and (b) proportion of species threatened divided by marine ecoregions.

Regarding the distribution of the assessed reef-building corals, most species are concentrated in the WC (68 species in total) (Fig 3). The Greater Antilles has the most corals with 63 species, followed closely by surrounding ecoregions (e.g., Southern Caribbean & Southwestern Caribbean). Of the 68 coral species in the WC, 51 are endemic (i.e., 75% of the coral diversity in the WC). Guianan ecoregion is found to have 59 species, but this is likely due to the proximity to the Southwestern Caribbean and so many species’ distribution maps are marked in ArcGIS as occurring in Brazil. The Brazilian province has the second highest number of corals across its ecoregions (23 species; nine endemics representing 13% of Brazil’s diversity) with Northeastern Brazil acting as the local diversity hotspot (23 species). The Eastern Atlantic has an overall lower number of corals (14 total; three endemics which represent 21% of the Eastern Atlantic’s diversity), with most eastern species found off the African coast in ecoregions such as the Gulf of Guinea Central (12 species). The Mediterranean Sea only has four species known to occur throughout all its ecoregions, with three of them endemics. It must be highlighted that the only reef-building coral in this sea (*C*. *caespitosa*) is listed as threatened.

The WC hosts the highest number of threatened species as seen in the Southern Caribbean and Southwestern Caribbean (28 species), but proportionally has an overall lower percentage given higher species richness. Brazilian ecoregions host the second highest number of threatened species, with most of the regionally threatened species found in Northeastern Brazil (13 species). Overall, the Brazilian province has a high percentage of at-risk species and contains the ecoregion with the highest ratio of threatened species, Trindade and Martim Vaz Islands with 62.5% of corals (five of eight species). There is a relatively low number of threatened corals found to occur within the Eastern Atlantic ecoregions, as seen in the Cape Verde and Azores Canaries Madeira (one species each). The low species richness, however, contributes to a high percentage of at-risk species in the Mediterranean, with 50% (two of four) of species threatened. The West African coast has the lowest percentage of threatened species within the Atlantic but the highest percentage of those classified as DD. In the Gulf of Guinea, 50% (three of six species) of corals are listed as DD.

### Annual severe bleaching

We highlight that most coral species found to be highly vulnerable to bleaching under both emission scenarios (SSP5-8.5 representing current global emissions and SSP2-4.5 representing future reductions in emissions) (Table 1) qualified for CR status. Exceptions were made for species whose dominant depth range exceeds 30 m from the surface, as a proportional decline was calculated from the assumed minimum decline of 80% due to the onset of ASB. On average, under SSP5-8.5, ASB is expected to occur by 2030 (not considering the possibility of adaptation) and under SSP2-4.5 this is only extended by three years (2033). This was for all the species that the projections were applied to. With only a three-year delay in the onset of ASB between scenarios, even the reduced emission scenario is well within the three-generation length time frame (30 years). In fact, both scenarios predict the onset of ASB within the time frame of a single generation.

### Coral cover

Seven species qualified for NT status based on the GCRMN coral cover data (*Agaricia grahamae*, *Madracis formosa*, *Mussa angulosa*, *Orbicella franksi*, *Porites branneri*, *Pseudodiploria clivosa*, and *Stephanocoenia intersepta*). A single species (*Millepora striata*) qualified for VU status under both the GCRMN and IPCC approaches. Overall, species rich ecoregions that represent large proportions of species’ total ranges have estimated positive trends in coral coverage (especially for the median estimate and 20% CI). For example, the Greater Antilles Ecoregion has an average +34.28% coral cover change over the past 30 years, while also representing the highest number of extant corals. Therefore, when using coral cover estimates, many species are estimated to have increases in population.

Yet, the reported changes in population are likely overestimations as minimum convex polygons (including marine area extending up to 200km from the coastline) were utilized to create species ranges, rather than species area of occurrence. For most of the species, occurrence data are not sufficient to calculate the area of occurrence, so minimum convex polygons provide a consistent means to measure species range and assess sub-regional losses of habitat [48]. Additionally, known reef layers fail to account for suitable habitat outside of reefs and cannot be used to create range maps for species in regions lacking coral reefs, such as the West African coast and the Mediterranean Sea.

Estimated coral cover change for several ecoregions had not been calculated due to the lack of sufficient coral cover data compiled by the GCRMN. These include the northeastern coast of the United States, southern Brazil, mid-Atlantic islands, and the entirety of the West African coast and Mediterranean Sea. For these ecoregions that may be at the margins of coral reef distribution, coral cover change was assumed to be zero. In doing so, the decline estimates for species with a large proportion of their range within these marginal areas may not accurately reflect total decline nor their true extinction risk.

### Back-casting to 2008 and comparison to 2021

**Fig 4:**
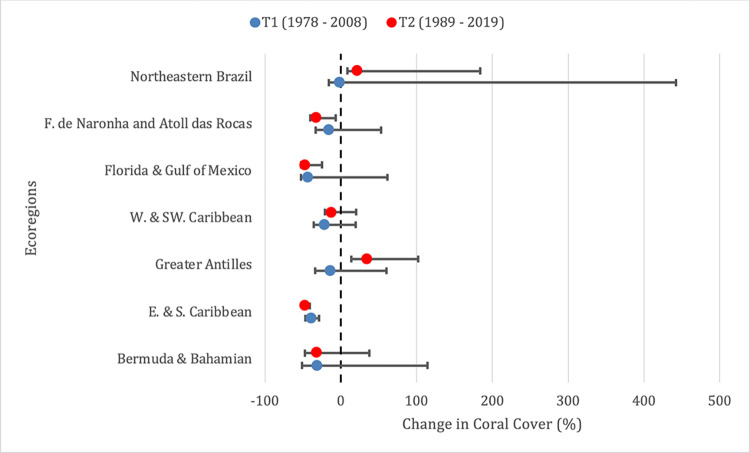
Estimated change in coral cover by marine ecoregions. Range in estimated change in coral cover by ecoregions of the Atlantic Ocean as modeled by the Global Coral Reef Monitoring Network for the T1 time-series (1978 – 2008; blue) and for the T2 time-series (1989 – 2019; red). Estimated changes in coral coverage include the median estimate (50%) as well as the 80% and the 20% uncertainty bounds or confidence intervals available for each ecoregion. Data points are not plotted for Eastern Brazil & Trindade & Martim Vaz Islands due to extremely high estimated changes in coral cover.

In comparing the time-series 1978 - 2008 (*T*_1_) with 1989 - 2019 (*T*_2_) from the eight Atlantic subregions with available GCRMN estimates, *T*_1_ depicts greater losses in coral cover in species rich ecoregions (i.e., Greater Antilles and Western and Southwestern Caribbean) (Fig 4). In contrast, *T*_2_ estimates more gains in coral coverage, that are applicable to species determined to be moderately and highly susceptible to decline, whereas gains in *T*_1_ are applied solely to more resistant species. It is likely that severe losses in the 1980s displayed a more drastic reduction in live coral cover that has yet to be rivaled in more recent decades, as hypothesized in parts of the Indo-Pacific [69]. The estimated gains in coral cover for select regions may point towards species adaptation to reoccurring threats (i.e., thermal stress and disease), environmental filtering of more susceptible species, or the alleviation of some local threats (i.e., coastal runoff, eutrophication, overfishing, etc.). However, the role that continuously rising SST, as modeled by the IPCC, will have on this trend remains uncertain.

If the GCRMN data were available during the 2008 assessments and used to categorize species, 32.9% (26 of 79 species) of corals would have qualified for a higher extinction risk than they were assigned. The number of changes would have been equal to those reported for this current update. Only 2.5% (two of 79) would have qualified for a lower extinction risk, and 39.2% (31 of 79) would have remained in the same category. However, the changes in status do not necessarily reflect the same finalized categorization identified in this current update. For example, *Agaricia grahamae* (originally LC) would have qualified for VU status using the *T*_1_ time series (based on a decline of -41.45% coral cover), but currently qualifies for NT status using the *T*_2_ time series (based on a decline of -25.45% coral cover). Overall, the GCRMN data suggest that loss of coral cover was more significant in *T*_1_ when compared to the *T*_2_ time series and is reflected in the threatened status of Atlantic corals, which would have been more threatened in 2008 compared to this current update.

## Discussion

Using the data-driven methodology applied in this study, the best estimate for the percentage of threatened Atlantic shallow water corals is approximately 50% (45.88% - 54.12%). According to this reassessment, a genuine change in status was only reported for one species - *Dendrogyra cylindrus* (VU to CR). A result which was based on observations of severe decline due to the recent spread and impacts of SCTLD on this species [70]. All other reported changes in status are considered non-genuine due to differences in methodologies between assessments. Thus, these results cannot accurately reflect realized deteriorations or recoveries of species population but rather differences in modeled data, which will need to be communicated appropriately depending upon the application of results for different policies and conservation actions.

To address this and to try and track changes in Red List status and identify potential ‘genuine’ changes, we performed a back-casting exercise to compare current decline estimates with those of 2008 using the same datasets. Doing so contributes to the Red List Index, which tracks changes in species status over time, highlighting marked improvements or deteriorations in extinction risk. Which in turn, better supports policies and management plans protecting at-risk species [46,71,72]. However, we were unable to fully replicate our methods as the UNEP (2020) projections only go back to 2015.

Recognizing that we cannot compare our data to the 2008 assessment, we still hold true to the data underpinning these assessments are the most up-to-date estimations of rising SST and changes in coral cover in the region [11,52]. Further, we also illustrate that via both the IPCC and GCRMN models utilized, an alarming number of Atlantic corals are listed as threatened. Moving forward we now have access to these freely available datasets for future reassessments and the overall identification of potential biodiversity loss.

For this assessment process, such datasets were also combined with a trait-based approach to estimate individual species’ vulnerability and resiliency to decline. Most studies on the loss of corals typically focus on the severity of coral reef loss at either regional or global levels [20,21,33,61,73–75]. While insights at the biome/ecosystem level are important for corals, changes in species composition are more relevant for measures of biodiversity and may be of higher priority to management/conservation plans [76]. Individual coral species differentially contribute to reef function, vary in their relative abundance, and respond to environmental stressors at differing degrees [41,77]. For example, in WC the abrupt coral die-off following the SCTLD outbreak disproportionally reduced the abundance of species that are key providers of three-dimensionality, shifting to the dominance of assemblages dominated by taxa with simpler morphological attributes and slower growth rates [28]. Understanding how each species has responded or is likely to respond to certain threats can provide a better understanding of shifts in population and ecosystem functionality [78].

### Species traits

The use of species-specific biological and ecological traits functioned to differentiate levels of vulnerability and resiliency to past and future threats [79]. This builds off the work of other studies that based species risk on differences in exposure, sensitivity, and adaptability [42,44]. However, as is common in trait-based approaches, gaps exist in the trait data for many species as well as for certain sets of traits. This was particularly the case for Eastern Atlantic (e.g., *Oculina patagonica*) species. SCTLD vulnerability had the most data gaps, given it is a relatively new disease and studies on its severity are not comprehensive for all corals [54]. Other traits, such as disease and bleaching vulnerability in general, are predominantly informed by expert opinion due to the lack of standardized measures of species susceptibility and are more subjective reflections of risk. In many cases, traits were treated as unknown values and received the middle trait rank of two (i.e., moderately susceptible), given the uncertainty in how the vulnerability compares to other corals.

Trait-based approaches are highly sensitive to the quality of trait information available, the number of traits considered, and how species are grouped [80]. Our approach utilized ten trait-based indicators of vulnerability and resiliency, including both continuous and categorical data divided amongst three levels of risk. By filling in missing traits information, including additional traits (e.g., pollution vulnerability), and/or altering grouping structure (i.e., modified thresholds, increased number of ranks, etc.), there is potential to alter species risk level. Additionally, we did not differentially assign weights to traits based on the level of impact or potential threat. It can be argued that bleaching vulnerability plays a larger role in population reduction compared to other traits, considering rising SST [81]. However, to what extent this is true relative to all threats remains unclear given the spatial and temporal variability of threats [45].

Moving forward, the usefulness of trait-based approaches (as utilized here) will benefit from more comprehensive studies on the response of species to known and potential threats that emphasize relevant trait characteristics [57,82]. Such work should consider both interspecies variation amongst different taxa, as well as intraspecies variation within populations. Outlining relative vulnerability and resiliency (comparing species), or specified thresholds of risk (limits of survival), can better equip trait-based approaches to accurately differentiate more at-risk from highly resistant species.

### Coral cover

The ten trait-based indicators resulted in the final rankings (3-highly susceptible, 2-moderately susceptible, and 1-more resistant) that ultimately determined the assigned level of decline (80%, 50%, and 20% CIs respectively) - as modelled by the GCRMN’s estimated changes in coral cover. The loss of live coral cover has served as a measure of habitat decline and ecosystem state in other studies [1,63]. Given that such data provide a poor proxy for species-level declines, species traits were used to distinguish those likely to have experienced severe losses in their population compared to more resistant corals.

Yet, geographical differences in baseline conditions and community structure are important factors when assessing population decline [83]. We assumed the populations at the ecoregion level were proportional to species’ total range. However, corals are not distributed evenly across reefs with various species serving as either the dominant reef-builders or rare species across sites with shifts in community structures commonly occurring after disturbances [23,77,84]. Some species may even increase with an overall decline in coral cover, due to reduced competition, faster rates of reproduction and growth rates, and or higher resiliency becoming increasingly more common when compared to historic community assemblages [84,85]. Thus, species traits serve as indicators of species vulnerability and resiliency, but still provide a poor proxy to true long-term population data across a species range.

For the various ecoregions, the GCRMN concludes that the WC is undergoing a large-scale transition towards an algal-dominated state, with Brazil seeing a more heterogenous relationship between live coral cover and algal cover [11]. Yet, for both regions, increases in coral cover are reported to be occurring. Especially in regional species diversity hotspots (i.e., Greater Antilles and Northeastern Brazil). In these cases, there is also an apparent stabilization in algal cover over the last few decades [11]. That said, it is highly unlikely that these ‘positive trends’ in live coral cover are even for all species. More data on community structure and species richness and evenness at all scales can better inform both species range maps and population models to improve our reflection of changes over time.

### Climate change and the interacting effects on coral

Only two trait-based indicators (bleaching vulnerability and dominant depth range) were ultimately used to inform species climate susceptibility to changing SST as modeled by the IPCC CMIP6 projections. Species ranked more resistant and moderately susceptible (rank 1 and 2 respectively) to bleaching susceptibility and assumed to be capable of adapting to 1°C above the maximum monthly mean, saw as much as a 30-40-year delay in the onset of ASB when compared to highly susceptible (rank 3) species. For species with a rank of 1 or 2, ASB is projected to occur in 2059 under SSP5-8.5 and 2073 under SSP2-4.5 (as opposed to 2030 and 2033 respectively for highly susceptible species). Of note, is that again both timelines are beyond the three-generation length time frame. The several decades delay in the onset of ASB for rank 1 & 2 species highlights both the value of a small degree of adaptation and the large discrepancies that are created from using such a model [86,87].

Given that the IPCC climate projections include quarter degree intervals between 0 – 2°C, there is potential to account for adaptation at a finer scale based on variation in the adaptability of species. However, with no metric to rank corals on bleaching susceptibility to this degree (related to the variation in interspecies adaptive potential), it remains challenging to assign species a level of adaptability that likely reflects their relative risk. We therefore had to assume a potential for adaptation given evidence to suggest corals are capable of acclimatizing and adapting to rising temperatures [56,65]. Nevertheless, there remains a need for a more comprehensive understanding of the factors that contribute to coral adaptation to changing climatic conditions and how this varies across biological and geographic scales [88].

Simultaneously, the dominant depth range was used to account for potential depth refugia for corals that are predominately found in deeper cooler waters and the 30 m depth limitation of the IPCC projections. Thus, species with wide depth ranges were estimated to experience reduced declines, assuming the potential for refugia from thermal stress in waters deeper than 30m [66,89]. However, the possibility for deeper waters to function as climate refugia remains contested [90]. Corals experiencing thermal stress at shallower depths may also experience it at deeper depths [67,68]. Therefore, again there is a degree of uncertainty in the decline estimates for corals with particularly wide depth ranges.

In a sensitivity analysis (comparing the assumed 80% decline as occurring at the predicted onset of ASB to that of the alternate threshold of the first instance of two bleaching events per decade), we found there to be no cases where changes in extinction risk status were needed. This is because, on average, two bleaching events per decade occur roughly four to seven years before the onset of ASB. Given that the average predicted onset of ASB for the Caribbean is 2033, either estimate of an 80% decline (e.g. onset of ASB or onset of two bleaching events per decade) occur well within the three-generation length time period (e.g. before 2050).

Regionally, the IPCC projections predict highly unsuitable conditions for Atlantic coral reefs, with modeled elevated SSTs seeing an on average onset of ASB by 2030. Even the ‘top refugia’ identified for the region (the Bahamas and the US-Florida & Texas) are still projected to see ASB after 2044, thus placing corals at risk within the next three-generations [52]. This amount of consistent widespread thermal stress will have ecosystem level impacts that go beyond inducing ASB events [56,91]. While the impact of climate change will no doubt be severe, the inclusion of species adaptation remains underrepresented in projected climate outcomes [92–94].

## Conclusions

A major challenge for coral species assessments is the lack of species-specific population data [44,50] alongside the challenges in coral species delimitation and current instability in their taxonomy [95–97]. Comparing relative extinction risk amongst species is aided by the availability of documented changes in populations. In the absence of such data, assessments must rely on modeled, inferred, or projected changes in populations [45]. Modeled data introduce uncertainty and makes it difficult to compare assessments that estimate declines differently [72]. Subsequently, comparing the severity of decline amongst species becomes challenging, especially when a clear metric is lacking. Species traits served as the means to distinguish more resilient corals from vulnerable species but was impeded by a lack of species data. The degree of uncertainty in these assessments emphasizes the need for standardized methodologies and the importance of filling the gaps in species-specific data. More research is needed on understanding species’ boundaries, adaptability to threats, populations and community dynamics, and documenting trait characteristics for all corals as well as the resolution of taxonomic uncertainty for the DD species (*Millepora laboreli*, *Oculina robusta*, *Oculina tenella*, *Oculina valenciennesi*, *Porites gabonensis*, *Schizoculina africana*, and *Schizoculina fissipara*).

Considering the threats facing coral reef ecosystems, the importance of continued monitoring, protection, and conservation efforts only become more vital. Globally, considerable progress needs to be made to climate policy as even the reduced emissions scenario (SSP2-4.5) projects the regional onset of annual severe bleaching events within the next decade. Yet, there might still be potential for adaptation to warmer waters (both at the species and ecosystem level), but this potential may be offset by more local perturbations. Mitigating local threats is especially valuable for the WC as it undergoes a continued transition towards an algal-dominated state. The protection and restoration of key herbivores (i.e., *Diadema* and parrotfishes), the management of pollutants and fishing practices, and the establishment and enhancement of marine protected areas (MPAs) could help reverse this trend and further encourage gains in coral cover as has been reported in some localities (e.g., Northeastern Brazil) [11]. Future reefs may see shifts in coral communities as species seek suitable habitat in response to climate change [98–101]. Protecting suitable waters outside of reef habitat or low-density reefs may increase the survivability of species as they migrate and/or adapt to changing SST. With the uncertainty in species potential for adaptation, maintaining biodiversity serves as an important safety net in ensuring climate-resilient reef systems.

## Supporting information

S1 Dataset Complete Atlantic coral species & assessors list. Dataset used to compile all relevant information used to inform species assessments including taxonomic information, trait rankings, year of annual severe bleaching onset for both CMIP6 SSP2-4.5 and SSP5-8.5 for degree intervals 0 and 1 based on species distributions, and percent decline as calculated from GCRMN data for the 20, 50, and 80 percentiles based on species distributions including backcasted declines for comparison to 2008 assessment results. Included is a full list of associated assessors and their affiliations.
(XLSX)
